# Reading fluency as the bridge between decoding and reading comprehension in Chinese children

**DOI:** 10.3389/fpsyg.2023.1221396

**Published:** 2023-08-29

**Authors:** Lucy Shih-Ju Hsu, Kevin Chan, Connie Suk-Han Ho

**Affiliations:** ^1^Department of Psychology, The University of Hong Kong, Pok Fu Lam, Hong Kong SAR, China; ^2^Centre for Child and Family Science, The Education University of Hong Kong, Tai Po, Hong Kong SAR, China

**Keywords:** reading fluency, Chinese reading, simple view of reading, reading comprehension, structural equation modeling

## Abstract

**Purpose:**

Reading fluency has been considered an essential component of reading comprehension, but it is yet to be examined in a reading model in a non-alphabetic writing system. This study investigated whether reading fluency could be identified as a separate construct from decoding and examined the unique role of reading fluency in the Simple View of Reading (SVR).

**Method:**

A total of 342 Cantonese-speaking Chinese children in grades 3–5 were recruited to participate in the study. They were assessed on word reading accuracy and fluency, morphological awareness, vocabulary knowledge, and reading comprehension.

**Results:**

The confirmatory factor analysis results confirmed that reading fluency is a separate factor from decoding, linguistic comprehension, and reading comprehension. Furthermore, the structural equation modeling results revealed that reading fluency is a significant predictor of reading comprehension and a mediator between decoding and reading comprehension in the extended SVR model.

**Conclusion:**

The findings extended previous research in alphabetic languages and supported reading fluency as the bridge between decoding and reading comprehension. The present study highlighted the importance of reading fluency in Chinese reading acquisition in a theoretical framework.

## Introduction

The Simple View of Reading (SVR) states that reading comprehension is the product of two key factors: decoding (D), i.e., the ability to recognize and pronounce words, and linguistic comprehension or language comprehension (LC), i.e., understanding the meaning of words and sentences, (D x LC = RC; Gough and Tunmer, 1986; Hoover and Gough, 1990). While the SVR has been validated by over 150 studies across different languages with different orthographic depths (see Florit and Cain, 2011 for a meta-analysis), whether this model fully captures the components needed for successful reading comprehension remains to be determined (Sparks, 2021). Reading fluency is conceptualized as the oral translation of the text with speed and accuracy (Fuchs et al., 2001), which is often operationalized as 1-minute fluency tasks requiring participants to read aloud a word list or a passage composed of high-frequency words. Based on the original SVR, studies in alphabetic languages have proposed the extended SVR, suggesting that in addition to decoding and linguistic comprehension, reading fluency may be a unique contributor to children's reading comprehension (Joshi and Aaron, 2000; Torgesen and Hudson, 2006; Hudson et al., 2008; Rasinski et al., 2011; Silverman et al., 2013; Torppa et al., 2016; Cadime et al., 2017; Kuhn and Schwanenflugel, 2019). However, the unique contributing role of reading fluency in non-alphabetic languages, for example, Chinese, is yet to be examined. The present study examined the role of reading fluency in reading comprehension in Chinese under the extended SVR framework.

### Reading fluency as an additional component to the extended Simple View of Reading

The Simple View of Reading (SVR), i.e., D x LC = RC, suggests that decoding is a “bottom–up” skill-based component and linguistic comprehension is a “top–down” cognitive-based component in reading comprehension (Gough and Tunmer, 1986; Hoover and Gough, 1990). Linguistic comprehension involves understanding the meaning of words in contextualized sentences, which relies on the reader's prior semantic knowledge. Linguistic comprehension is usually measured using morphological awareness, oral vocabulary knowledge, and listening comprehension (Adlof et al., 2006; Tilstra et al., 2009), which is generally undisputed. Decoding emphasizes the accuracy and pattern recognition of letters and words. Studies, however, have employed different operationalizations of decoding in SVR. Some measured decoding using reading accuracy tasks in which participants are asked to read aloud a word list in the ascending order of difficulty levels without a time limit (Joshi and Aaron, 2000; Georgiou et al., 2009), while others have combined reading accuracy and fluency measures to a single variable (Adlof et al., 2006; Kirby and Savage, 2008; Kershaw and Schatschneider, 2012).

Reading fluency is an automatic-activation process, encompassing perceptual skills and a print-to-sound translation process, making it more complex than a pure decoding measure (Fuchs et al., 2001; Wolf and Katzir-Cohen, 2001). Researchers have suggested that fluency might play a more prominent role in later grades in readers of opaque orthographies due to the demand for sound-print mapping as compared to readers of transparent orthographies (Wimmer et al., 1998; Aaron et al., 1999; Florit and Cain, 2011). According to the verbal efficiency theory (Perfetti, 1985), decoding must become automatic to allocate cognitive resources for reading comprehension. Reading fluency has been found to significantly predict reading comprehension (Cutting and Scarborough, 2006; Silverman et al., 2013; Torppa et al., 2016). Silverman et al. (2013) examined the role of reading fluency in a group of fourth-grade students in the United States. Their results showed that reading fluency was a unique predictor of reading comprehension, which mediated the prediction of decoding and linguistic comprehension on reading comprehension. Torppa et al. (2016) and Cadime et al. (2017) found similar results in a sample of Finnish kindergarteners to grade 3 and Portuguese grade 2 students, respectively. Studies conducted on Korean kindergarteners and grade 1 students have shown that reading fluency is significantly associated with reading comprehension above and beyond decoding and linguistic comprehension in the extended SVR framework (Kim et al., 2014; Kim, 2015). In line with Florit and Cain's meta-analysis (2011), reading fluency is a significant predictor of reading comprehension across transparent (Finnish: Torppa et al., 2016), intermediate (Portugal; Cadime et al., 2017), and opaque (English: Silverman et al., 2013) orthographies as well as languages in logographic shape (Korean: Kim et al., 2014). The Chinese language, however, lies in the further ends of the opaque orthographic consistency spectrum. Previous studies have not examined whether reading fluency plays a unique role in reading comprehension under the extended SVR. The present study aimed to fill this literature gap by examining the role of reading fluency in reading comprehension under the extended SVR in a sample of Chinese primary-grade students.

Furthermore, previous studies have identified the mediating role of reading fluency in the extended SVR (Silverman et al., 2013; Torppa et al., 2016; Cadime et al., 2017). Based on the verbal efficiency theory (Perfetti, 1985), researchers examined whether reading fluency mediates the relationship between decoding and reading comprehension. In the longitudinal study by Cadime et al. (2017) in a sample of Portuguese grade 2 students, the results showed a significant mediating role of reading fluency between decoding and reading comprehension in grades 2 and 4. Moreover, Silverman et al. (2013) found that reading fluency is a significant mediator between decoding, linguistic comprehension, and reading comprehension. The results supported the meta-analysis by Florit and Cain (2011) that orthographic transparency might influence the role of reading fluency in the extended SVR. In light of these diverse findings, studies in Chinese have included reading fluency in the SVR (Ho et al., 2017; Yan et al., 2021; Pan and Lin, 2022) in primary-grade children. None of them, however, have considered the unique mediating role of reading fluency in the extended SVR. Despite growing evidence that reading fluency might be a separate measure, reading fluency has been consistently considered a measure of decoding. Therefore, this study examined the factor structures of decoding and reading fluency using confirmatory factor analysis and the unique predictive role of reading fluency in the extended SVR using structural equation modeling.

### Reading acquisition in Chinese

As a more opaque language, Chinese has a morpho-syllabic writing system (DeFrancis, 1984). Unlike alphabetic languages, Chinese characters (the smallest unit of Chinese words) are more visually complex. Each character represents a morpheme and syllable (DeFrancis, 1984; Hoosain, 1991). Over 80% of Chinese characters consist of semantic and phonetic radicals. Semantic radicals carry the meaning of the character, while the phonetic radical provides the phonetic cue of the character although the phonetic cue is far from reliable. The abundance of homophones and homographs in Chinese might further complicate the reading process. Chinese readers must attend to contextual information in sentences or passages to disambiguate the meaning of words. Moreover, the lack of boundaries between characters might put an excessive cognitive load on Chinese reading comprehension (Liu, 2014). Given the less reliance on phonetic cues in Chinese reading, although phonological awareness plays a significant role in Chinese reading, it reaches a ceiling effect in an early stage of reading development. By contrast, other cognitive-linguistic skills, for example, orthographic knowledge, morphological awareness, and rapid naming, have been shown to be more important in Chinese children's reading development (McBride-Chang et al., 2003; Ho et al., 2004; Yeung et al., 2013).

Moreover, the learning and instruction of Chinese vary across different Chinese-speaking regions. Chinese readers from mainland China, whose official spoken language is Putonghua, learn to read Chinese through the pinyin phonetic coding system. However, no phonetic coding system is used by Chinese readers from Hong Kong, where Cantonese is the most common spoken language. Therefore, Hong Kong Chinese readers rely more on rote memory and lexical retrieval efficiency to learn to read Chinese. Furthermore, traditional Chinese characters are used in Taiwan and Hong Kong, which are more visually complex than simplified Chinese characters used in China. The differences in Chinese languages between regions might influence the relationship between cognitive-linguistic and literacy skills (McBride-Chang et al., 2005; McBride, 2015). The present study focused on Chinese children from Hong Kong who spoke Cantonese and wrote traditional Chinese characters as their mother tongue to examine the extended SVR in Chinese.

### The Simple View of Reading in Chinese

The SVR with reading fluency as a measure of decoding has been examined widely in Chinese, particularly in Cantonese-speaking children (Ho et al., 2012, 2017; Yeung et al., 2013; Pan and Lin, 2022). Yeung et al. (2013) examined the SVR in Chinese in a sample of grade 4 Cantonese-speaking children in Hong Kong. The results showed that reading comprehension was significantly predicted by decoding (measured by word reading accuracy) and syntactic skills (measured by word and sentence orders). Linguistic (measured by morphological awareness) and rapid naming did not significantly predict reading comprehension. Although rapid naming is a speeded measure, previous studies have shown that rapid naming is a significant predictor of reading fluency, which measures the lexical retrival speed (Georgiou et al., 2016).

Yeung et al. (2016) further examined the SVR in their longitudinal study of grade 1 Cantonese-speaking children in Hong Kong. Text-reading fluency and word-reading accuracy were measured as the decoding variable. Linguistic comprehension was measured by oral vocabulary, narrative, and syntactic skills. The results showed that in line with SVR, decoding and linguistic comprehension were significant predictors of reading comprehension. However, the influence of reading fluency was not examined independently. Furthermore, Ho et al. (2017) examined the extended SVR by including rapid naming as a third predictor in a sample of grades 1 to 3 Cantonese-speaking children in Hong Kong. The confirmatory factor analysis showed a four-factor structure: rapid naming, decoding, linguistic comprehension, and reading comprehension, and that word-reading fluency regressed on both rapid naming and decoding. The structural equation model results showed that decoding and linguistic comprehension but not rapid naming were significant predictors of reading comprehension. Consistent with Yeung et al.'s (2013) findings, rapid naming might not be the proximal predictor of reading comprehension; instead, rapid naming is possibly a distal predictor *via* decoding (Yeung et al., 2013; Ho et al., 2017). Therefore, rapid naming was not included in this study. Since the role of reading fluency in the extended SVR in Chinese is still underexplored, the present study aimed to examine the proximal variables of reading comprehension based on the extended SVR: decoding, linguistic comprehension, and reading fluency.

### The present study

Findings from previous studies in Chinese reading have suggested that decoding and linguistic comprehension play important roles in SVR. Despite the plethora of studies on Chinese reading, very few examined reading fluency as an independent measure in a Chinese reading comprehension model. Furthermore, of those that included reading fluency, the measure was only examined at either word (Ho et al., 2017) or text level (Yeung et al., 2016) and was submerged into decoding. Therefore, the present study aimed to examine the unique role of reading fluency, measured by word and text-level measures to understand Chinese reading comprehension better based on the extended SVR.

The first aim of the present study was to investigate whether reading fluency, measured by word- and text-reading fluency, could be a separate construct from decoding using confirmatory factor analysis (CFA). Some studies have considered reading fluency a separate variable from decoding (Torgesen and Hudson, 2006; Silverman et al., 2013). On the other hand, some research studies have considered reading fluency to measure decoding skills (Yeung et al., 2016; Ho et al., 2017). Based on the verbal efficiency theory (Perfetti, 1985), we expected that reading fluency would be a separate factor from decoding, such that a four-factor model, reading fluency, decoding, linguistic comprehension, and reading comprehension, would fit the data better than a three-factor model, decoding + reading fluency, linguistic comprehension, and reading comprehension.

The second aim was to elucidate the unique role of reading fluency in the extended SVR model in Chinese reading through structural equation modeling (SEM) analysis. Guided by previous studies (Cutting and Scarborough, 2006; Silverman et al., 2013; Torppa et al., 2016), we expected that reading fluency would be a unique predictor of reading comprehension. Moreover, reading fluency would significantly mediate the relationship between decoding and reading comprehension. By achieving these objectives, we expect to understand better the unique role of reading fluency in the extended SVR in Chinese reading.

## Methods

### Participants

Participants were 342 Cantonese-speaking Chinese children in grades 3–5 (*M*_age_ = 10.04 years; *SD*_age_ = 0.98 years; girls = 163) from three primary schools in Hong Kong. Children were recruited by sending invitation letters to the parents of all children in grades 3–5 from the participating schools. All the children were screened to identify typically developing Chinese children without learning disabilities or sensory impairments.

### Procedure and materials

Written informed consent was obtained from the parents of the children before proceeding with the study. All measures were administered to individual children in a 45-min session by trained research assistants in a quiet room at the schools.

#### Word-reading fluency

Modeled after Ho et al.'s (2000) study, this task assessed children's reading speed of context-free words. Participants were asked to read aloud a wordlist of 110 high-frequency Chinese two-character words arranged in ascending order of difficulty as fast and as accurately as possible in 1 min. These words were selected from the Leixcal Lists for Chinese Learning provided the Hong Kong's Education Bureau for children in grades 1 to 3. One mark was awarded for each correctly read word. The reliability was calculated based on Gulliksen's formula for a time-limited task, *r*_lower_ =0.76 (Chan and Yeung, 2020).

#### Text-reading fluency

Modeled after Ho et al.'s (2000) study, this task assessed children's reading speed of connected text. Participants were asked to read aloud a passage of 310 Chinese characters as fast and accurately as possible in 1 min. These words were selected from the Lexical Lists for Chinese Learning for children in grades 1 to 3. One mark was awarded for each correctly read character. The reliability was calculated based on Gulliksen's formula for a time-limited task, *r*_lower_ = 0.90 (Chan and Yeung, 2020).

#### Character reading

The character reading task assessed children's decoding skills of single Chinese characters. Children were required to read aloud a wordlist of 60 Chinese characters arranged in ascending order of difficulty. The characters were selected from the Lexical Lists for Chinese Learning for children in grades 2 to 6. One mark was awarded to each correctly read character. The maximum possible score was 60, and Cronbach's alpha was 0.94.

#### Word reading

Modeled after Ho et al.'s (2000) study, this task assessed children's decoding skills of Chinese words. Participants were required to read aloud a wordlist of 150 Chinese two-character words arranged in ascending order of difficulty. These words were selected from the Lexical Lists for Chinese Learning for children in grades 2 to 6 and were non-existent in the character reading task. One mark was awarded to each correctly read a word. The maximum possible score was 150, and Cronbach's alpha was 0.96.

#### Vocabulary knowledge

This task was modified after McBride-Chang et al.'s (2008) study and assessed children's vocabulary depth and breath. For each trial, participants were required to provide an oral explanation of a Chinese word, for example, “公平” (fair), an explanation would be “平等” (equal) or “公正” (just). This task comprised 16 Chinese words selected from the Lexical Lists for Chinese Learning. Two points were used to score participants' answers based on the clarity of explanations. The maximum possible score was 32, and Cronbach's alpha was 0.70.

#### Morphological awareness

Modeled after Liu and McBride-Chang's (2010) study, this task assessed children's ability to morpheme a construction. For each trial, participants were required to construct a new Chinese word based on a two-sentence scenario, for example, “用手開嘅槍叫手槍, 用腳開嘅槍我哋會點叫佢呀?” (A gun that is triggered by hand is called a handgun. If there is a gun that is triggered by foot, what do we call it?), and the correct answer was “腳槍” (foot gun). This task consisted of 38 items. One mark was awarded for each correctly produced word. The maximum possible score was 38, and Cronbach's alpha was 0.71.

#### Sentence comprehension

This task assessed children's sentence comprehension skills. Participants were required to read a sentence silently and answer one multiple-choice question. This task consisted of 20 sentences and multiple-choice questions. One point was awarded for each correct answer. The maximum possible score was 20, and Cronbach's alpha was 0.76.

#### Passage comprehension

The passage comprehension task was used to assess children's reading comprehension skills. Participants were required to read two narrative passages silently and answer six multiple-choice questions in each passage. These questions were designed to focus on (1) retrieval of information, (2) making direct inferences, (3) interpreting and integrating presented information; and (4) evaluating content, language, and contextual components, based on the Progress in International Reading Literacy Study (Mullis et al., 2007). One point was awarded for each correct answer. The maximum possible score was 12, and Cronbach's alpha was 0.67.

#### Raven's standard progressive matrices

Raven's standard progressive matrices were used as an intelligence assessment (Raven, 2003). For each item, children were presented with a visual stimulus with a missing part and were required to select the best matching piece out of six or eight options. This task consisted of 60 items. One mark was awarded for each correct answer.

### Data analysis

The full information maximum likelihood (FIML) was used to handle missing data (1.5% in this study). In addition, descriptive statistics and correlation after controlling for the grade level and IQ were performed for all measures. To examine whether reading fluency is a separate construct from decoding, a CFA using maximum likelihood was calculated to examine the three-factor (Model 1A) and four-factor (Model 1B) structure of reading fluency, decoding, linguistic comprehension, and reading comprehension. The original SVR model was tested to examine the validity of the SVR model in Chinese reading (Model 2). Moreover, three SEMs using maximum likelihood were conducted to examine the role of reading fluency in the SVR model in Chinese reading. Model 3A examined the unique role of reading fluency, Model 3B examined the mediating role of reading fluency between decoding and reading comprehension, and Model 3C examined the indirect effect of reading fluency on reading comprehension via decoding. Furthermore, moderation analyses were conducted to examine whether the relationship between reading fluency and reading comprehension was moderated by the grade level and gender of participants.

Model fits were determined using multiple indices: χ^2^ test, root mean square of approximation (RMSEA), standard root mean residual (SRMR), comparative fit index (CFI), and Akaike information criterion (AIC). A non-significant χ^2^ value, RMSEA <0.08, SRMR <0.06, and CFI > 0.95, indicated a good fit to the data (Hu and Bentler, 1999). Model fits were compared using χ^2^ tests and AIC values, where a 2-unit less in AIC values indicated better model fits. The indirect and moderating effects were examined using bias-corrected bootstrap with 2,000 resamples. The significance of indirect effects was determined using *p*-values and 95% confidence intervals (CIs), where the 95% CI not passing zero indicates a significant indirect effect (Preacher and Hayes, 2008; Memon et al., 2018).

## Results

### Descriptive statistics and correlations

The means, standard deviations, and correlations after controlling for grade levels and IQ for all measures are shown in Table 1. Reading comprehension measures were significantly associated with all measures, *r*s ≥ 0.22, and significantly intercorrelated, *r* = 0.38. Reading fluency measures were significantly associated with decoding measures and linguistic comprehension measures, *r*s ≥ 0.24, and significantly intercorrelated, *r* = 0.82. Decoding measures were significantly associated with linguistic comprehension measures, *r*s ≥ 0.37, and significantly intercorrelated, *r* = 0.80. Linguistic comprehension measures were significantly intercorrelated, *r* = 0.38.

**Table 1:** Descriptive statistics and correlation after controlling for grade levels and IQ.

		**1**	**2**	**3**	**4**	**5**	**6**	**7**	**8**
1	Word-reading fluency								
2	Text-reading fluency	0.82							
3	Character reading	0.45	0.44						
4	Word reading	0.43	0.41	0.80					
5	Vocabulary knowledge	0.26	0.28	0.46	0.39				
6	Morphological awareness	0.24	0.32	0.46	0.37	0.38			
7	Sentence comprehension	0.43	0.44	0.65	0.61	0.40	0.42		
8	Passage comprehension	0.23	0.22	0.40	0.34	0.31	0.23	0.38	
	*M*	82.06	177.39	40.47	98.30	11.69	20.17	14.89	8.97
	*SD*	18.46	42.75	11.14	29.91	4.20	6.00	2.35	3.25

All correlation coefficients were significant at *p* < 0.001.

### Confirmatory factor analysis

**Figure 1:**
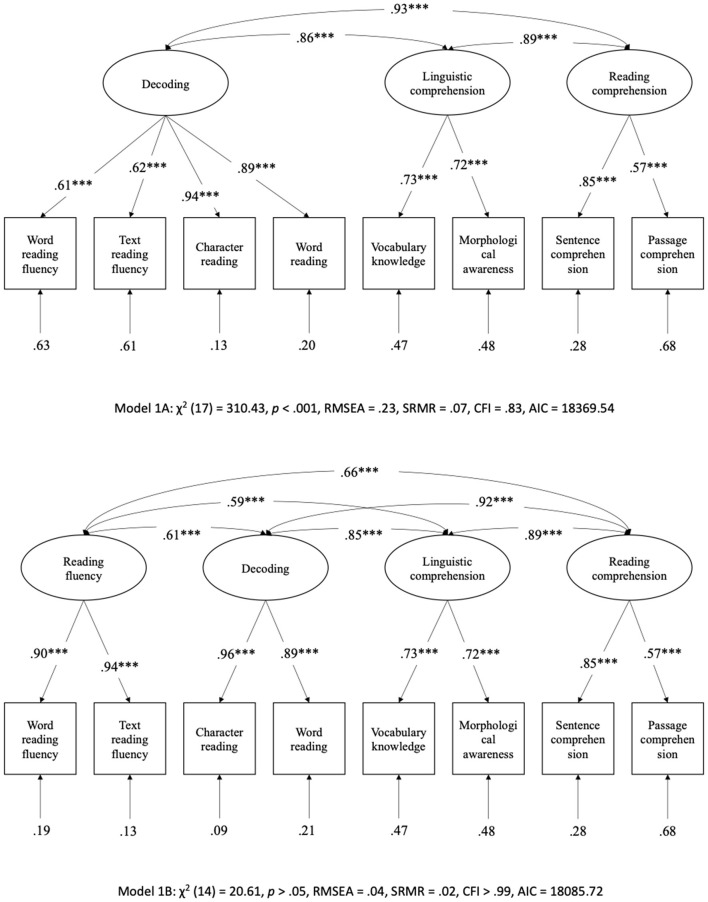
Confirmatory factor analysis examining the factors in the Simple View of Reading. ^***^p < 0.001.

As shown in Figure 1, Models 1A and 1B showed the three-factor and four-factor structure of the SVR in Chinese reading, respectively. Model 1A showed a poor fit to the data, χ^2^ (17) = 310.43, *p* < 0.001, RMSEA = 0.23, SRMR = 0.07, CFI = 0.83, and AIC = 18,369.54. Model 1B showed an excellent fit to the data, χ^2^ (14) = 20.61, *p* > 0.05, RMSEA = 0.04, SRMR = 0.02, CFI > 0.99, and AIC = 18,085.72. Moreover, the four latent variables were significantly associated, *r*s ≥ 0.59. This result supported our hypothesis that reading fluency is an additional and unique factor from decoding. Therefore, Model 1B was used for further analysis in this study.

### Structural equation modeling

**Figure 2:**
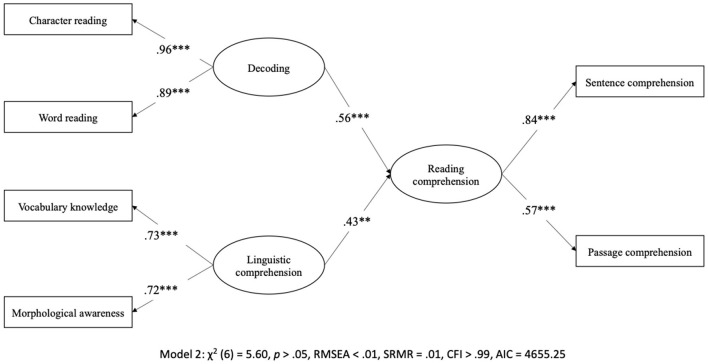
Structural equation modeling examining the original Simple View of Reading in Chinese. ^**^p < 0.01, ^***^p < 0.001. Covariances between independent variables were omitted for readability.

**Figure 3:**
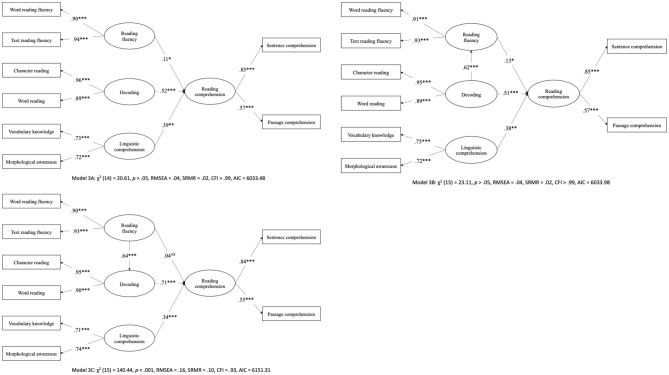
Structural equation modeling examining the role of reading fluency in the Simple View of Reading in Chinese. NS, Not significant, *p < 0.05, **p < 0.01, ***p < 0.001. Covariances between independent variables were omitted for readability.

Structural equation modeling (SEM) was used to examine the role of reading fluency in the SVR. We first examined the original SVR (Model 2) to confirm the relationship between decoding, linguistic comprehension, and reading comprehension in Chinese. As shown in Figure 2, Model 2 shows an excellent fit to the data, χ^2^ (6) = 5.60, *p* > 0.05, RMSEA <0.01, SRMR = 0.01, CFI > 0.99, and AIC = 4,655.25, supporting SVR framework as a Chinese reading model. Figure 3 shows Model 3A, 3B, and 3C examining the unique and mediating role of reading fluency in the SVR. Model 3A showed an excellent fit to the data, χ^2^ (14) = 20.61, *p* > 0.05, RMSEA = 0.04, SRMR = 0.02, CFI > 0.99, and AIC = 6,033.48. Reading fluency, β = 0.11, decoding, β = 0.52, and linguistic comprehension, β = 0.39, were significant predictors of reading comprehension. Model 3B also showed an excellent fit to the data; χ^2^ (15) = 23.11, *p* > 0.05, RMSEA = 0.04, SRMR = 0.02, CFI > 0.99, and AIC = 6,033.98. Reading fluency, β = 0.13, decoding, β = 0.51, and linguistic comprehension, β = 0.38, were significant predictors of reading comprehension. Moreover, decoding significantly predicted reading fluency, β = 0.62, and reading comprehension via reading fluency, β = 0.08, 95% CI [0.01, 0.15]. Model 3C, however, showed a poor fit to the data; χ^2^ (15) = 140.44, *p* < 0.001, RMSEA = 0.16, SRMR = 0.10, CFI = 0.93, and AIC = 6,151.31. Therefore, Model 3C was excluded from further analysis.

Because both Model 3A and 3B showed an excellent fit to the data, a model fit comparison was conducted for the selection of the preferred model in this study. Table 2 shows the model fitting results of the above-examined models. A comparison of the overall fit statistics and model paths between Model 3A and 3B did not show a statistically better model; Δχ^2^ = 2.50, Δ*df* = 1, *p* >0.05, and ΔAIC = 0.50. According to the verbal efficiency theory (Perfetti, 1985) and the hypothesis from Fuchs et al. (2001), reading fluency must be achieved to free up cognitive resources for reading comprehension, and hence, the development of reading fluency is preceded by decoding and mediating the relationship between decoding and reading comprehension. Therefore, based on the theory, Model 3B was preferred in this study, supporting the mediating role of reading fluency in the SVR.

**Table 2:** Model fitting results of the confirmatory factor analysis (CFA) and structural equation models of the Simple View of Reading (SVR).

**Model**	**Description**	**χ^2^**	**RMSEA**	**SRMR**	**CFI**	**AIC**
1A	Three-factor CFA model	310.43^***^	0.23	0.07	0.83	18,369.54
1B	Four-factor CFA model	20.61	0.04	0.02	>0.99	18,085.72
2	Original SVR model	5.60	<0.01	0.01	>0.99	4,655.25
3A	SVR model with reading fluency as a predictor	20.61	0.04	0.02	>0.99	6,033.48
3B	SVR model with reading fluency mediating the relationship between decoding and reading comprehension	23.11	0.04	0.02	>0.99	6,033.98
3C	SVR model with decoding mediating the relationship between reading fluency and reading comprehension	140.44	0.16	0.10	0.93	6,151.31

^***^p < 0.001.

Based on Model 3B, moderation analyses were conducted to examine the effect of grade level and gender in the path model. The results showed that the grade level did not significantly moderate the relationship between reading fluency–reading comprehension, β = −0.05, 95% CI [−0.18, 0.06], decoding–reading fluency, β = 0.03, 95% CI [−0.09, 0.14], decoding–reading comprehension, β = 0.02, 95% CI [−0.01, 0.04], and linguistic comprehension–reading comprehension, β = −0.01, 95% CI [−0.06, 0.05]. Moreover, the results showed that gender did not significantly moderate the relationship between reading fluency–reading comprehension, β = −0.09, 95% CI [−0.25, 0.10], decoding–reading fluency, β = 0.12, 95% CI [−0.06, 0.30], decoding–reading comprehension, β = 0.01, 95% CI [−0.04, 0.07], and linguistic comprehension–reading comprehension, β = 0.02, 95% CI [−0.07, 0.10].

## Discussion

The present study examined the distinctiveness of reading fluency from decoding and the role that reading fluency plays in the extended SVR model in Chinese children. The results of CFA supported the hypothesis that reading fluency is a separate factor from decoding. Moreover, SEM results revealed that reading fluency is a unique predictor of reading comprehension, mediating between decoding and reading comprehension.

### Reading fluency as a separated factor from decoding

Word reading and reading fluency are defined as reading accuracy and automaticity, respectively (Fuchs et al., 2001). However, previous studies in Chinese have consistently considered reading fluency as a measure of decoding skills rather than a separate measure (Yeung et al., 2016; Ho et al., 2017). Only one study examined the factor structure using CFA (Ho et al., 2017). Moreover, previous studies in Chinese have employed either word- or text-reading fluency and submerged reading fluency into decoding (Yeung et al., 2016; Ho et al., 2017). The present study is the first to examine the extended SVR in Chinese, employing word- and text-reading fluency measures simultaneously to represent the reading fluency domain and enhance the generalizability of the variable. In line with the extended SVR (Silverman et al., 2013), the results from the CFA showed that the four-factor model fitted the data better, suggesting that reading fluency is a separate skill from decoding, linguistic comprehension, and reading comprehension in the Chinese language.

Silverman et al. (2013) suggested that reading accuracy and fluency develop simultaneously in early childhood and independently in fourth grade and beyond. Participants in this study were in grades 3 to 5, consistent with Silverman et al.'s (2013) findings. Previous studies in Chinese reading have been conducted with children in grades 1 to 3 (Yeung et al., 2016; Ho et al., 2017); therefore, it is possible that the grade level of participants might influence the factor structure of reading fluency and decoding. The present study warranted that reading fluency is separated from word reading in the Chinese writing system under the extended SVR framework. In addition, compared to the previous studies in Chinese SVR, our results suggested that the independent development between reading accuracy and fluency is plausible after junior elementary years. Future longitudinal studies are needed to further explore the development of reading accuracy and fluency and their influence on reading comprehension in the extended SVR.

### Role of reading fluency in the Simple View of Reading

The present study is the first to examine the mediating role of reading fluency in Chinese reading. In line with the verbal efficiency theory (Perfetti, 1985) and previous findings of the extended SVR in alphabetic languages (Tilstra et al., 2009; Silverman et al., 2013), the present study filled the gap in the literature that reading fluency is an additional unique contributor to reading comprehension in Chinese and advancing the extended SVR in Chinese. Moreover, the results of the present study showed that reading fluency significantly mediated the relationship between decoding and reading comprehension.

According to the verbal efficiency theory (Perfetti, 1985), one must achieve automatic word decoding to allocate more cognitive resources for reading comprehension. Therefore, readers with faster word decoding, i.e., better reading fluency, would be better in reading comprehension. This result is in line with previous studies in English, Finnish, Portuguese, and Korean (Joshi and Aaron, 2000; Silverman et al., 2013; Kim et al., 2014; Torppa et al., 2016; Cadime et al., 2017). In addition, the present finding of reading fluency as the mediator between decoding and reading comprehension also echoes the study by Silverman et al. (2013) in English readers and supports Pikulski and Chard's (2005) proposition of reading fluency as the bridge for children to proceed from decoding at the word level to comprehension at the text level. The mediating role that reading fluency plays in the context of the Chinese language suggested a diminished effect of decoding in Chinese reading comprehension when reading fluency is accounted for, which is a similar pattern observed in English readers (Silverman et al., 2013). Once children are capable of automating the process of word recognition, their decoding skill is insufficient to support their comprehension performances. Instead, processing words and text in a timely manner is more important for children's reading comprehension. Relying more on reading fluency in reading comprehension enables children to transfer cognitive resources to other linguistic skills, such as inference and retrieval of past knowledge.

Finally, moderation analyses revealed that grade levels and gender did not significantly moderate the contribution of reading fluency, decoding, and linguistic comprehension on reading comprehension. Previous studies have shown that girls consistently perform better than boys in reading comprehension and read more books than boys in primary schools (Logan and Johnston, 2009, 2010). However, studies about extended SVR have not yet examined the stability of SVR across gender. The results of the present study showed that the extended SVR is stable across gender, where the paths do not differ between boys and girls. While the present study was not a longitudinal study, our moderation analyses revealed that the grade level was not a significant moderator of the present extended SVR model. The model remains significantly stable across intermediate grade levels.

### Limitations and future directions

There are several limitations to the present study. One caveat of this study was the lack of data on younger and beginning readers, hindering the extent of the present finding. As suggested by Silverman et al. (2013), the role of reading fluency becomes more salient after grade fourth, and there is a lack of empirical evidence to support that grade fourth is the transition period of the development of reading fluency. Therefore, future studies should consider comparing the role of reading fluency in the SVR between younger and older readers. Additionally, the construct of linguistic comprehension in this study did not include any listening comprehension measures, often included in studies of SVR models, to account for children's higher mental processes at the sentence and discourse levels. Future research studies should include listening comprehension and discourse measures at the text level to further unravel the components of reading comprehension. Another limitation was that the present research only examined oral reading fluency concerning rate and accuracy. Including measures of silent-reading fluency may also add additional variances to reading (Price et al., 2016). The developmental effect could not be examined in the present study without longitudinal follow-up to examine grade-level effects and the potential changes in the mediating relationship of the extended SVR model.

### Conclusion and implications

Despite the limitations, this study contributes to the field by extending the current understanding of SVR and reading fluency in Chinese children's reading development. The present study was the first to examine reading fluency thoroughly in the SVR model in a language with opaque orthography. The results of the present study extended the SVR and demonstrated that reading fluency, the ability to retrieve words quickly, could be a separate reading skill from decoding, which may play a unique role in reading comprehension and a bridging role between decoding and reading comprehension. In general, the findings underscore the critical role of reading fluency in children's reading development. Moreover, the results suggest the significance of incorporating reading fluency measures at various linguistic levels to obtain a comprehensive assessment of children's fluency performance. These findings hold important implications for education. It is recommended that classroom assessments and intervention programs consider reading fluency as a core component of the curriculum. By focusing on reading fluency, educators can better address the needs of children who demonstrate proficient decoding skills but continue to struggle with text comprehension. Intervention programs prioritizing reading fluency can facilitate children's reading development from decoding individual words to comprehending texts. In conclusion, this study provides valuable insights into the importance of reading fluency in the reading development of Chinese children.

## Data availability statement

The raw data supporting the conclusions of this article will be made available by the authors, without undue reservation.

## Ethics statement

The studies involving human participants were reviewed and approved by University of Hong Kong's Human Research Ethics Committee. Written informed consent to participate in this study was provided by the participants' legal guardian/next of kin.

## Author contributions

LH and CH contributed to the conception and design of the study. KC performed the statistical analysis and wrote sections of the manuscript. LH wrote the first draft of the manuscript. All authors contributed to manuscript revision, read, and approved the submitted version.
